# Does sodium bicarbonate based extra-cellular buffering support reduce high intensity exercise-induced fatigue and enhance short-term recovery assessed by selected blood biochemical indices?

**DOI:** 10.5114/biolsport.2024.125591

**Published:** 2023-05-25

**Authors:** Krzysztof Durkalec-Michalski, Joanna Kamińska, Bryan Saunders, Andrzej Pokrywka, Igor Łoniewski, Michal Steffl, Tomasz Podgórski

**Affiliations:** 1 Department of Sports Dietetics, Poznan University of Physical Education, 61-871 Poznan, Poland; 2 Sport Sciences–Biomedical Department, Faculty of Physical Education and Sport, Charles University, 162 52 Prague, Czech Republic; 3 Department of Physiology and Biochemistry, Poznan University of Physical Education, 61-871 Poznan, Poland; 4 Applied Physiology and Nutrition Research Group, School of Physical Education and Sport, Rheumatology Division, Faculdade de Medicina FMUSP, University of São Paulo, São Paulo, Brazil; 5 Institute of Orthopedics and Traumatology, Faculty of Medicine FMUSP, University of São Paulo, Brazil; 6 Department of Biochemistry and Pharmacogenomics, Medical University of Warsaw, 02-097 Warsaw, Poland; 7 Department of Biochemical Sciences, Pomeranian Medical University in Szczecin, 71-460 Szczecin, Poland

**Keywords:** CrossFit, Supplementation, Muscle damage, Heamatological markers, Sodium bicarbonate, Biochemical markers

## Abstract

Exercise-induced metabolic processes induce muscle acidification which contributes to a reduction in the ability to perform repeated efforts. Alkalizing agents such as sodium bicarbonate (NaHCO_3_) prevent large blood pH changes, however, there is no evidence on whether regulation of acid-base balance may also support whole body homeostasis monitored through heamatological and biochemical blood markers in a dose-dependent manner. Thirty Cross-Fit-trained participants were studied in a randomized, multi cross-over, placebo (PLA)-controlled double-blind manner in which they performed a control session (CTRL, without supplementation), three NaHCO_3_ visits (three different doses) and PLA (sodium chloride in an equimolar amount of sodium as NaHCO_3_). Each visit consisted of two 30-s Wingate tests separated by CrossFit-specific benchmarks (Wall Balls and Burpees – both performed for 3 min). Blood samples were collected at rest, immediately post-exercise and after 45 min recovery. Significant differences between visits appeared for blood pH, percentage of lymphocytes and granulocytes, red blood cells count and haemoglobin concentration at post-exercise and 45-min recovery, and for white blood cells count, percentage of monocytes, concentration of magnesium and creatinine at 45-min recovery. Most of the observed differences for heamatological and biochemical markers were significant compared to CTRL, but not different after PLA. NaHCO_3_ supplementation compared to PLA did not significantly affect exercise or recovery shifts in studied blood indicators. However, the changes in these markers after NaHCO_3_ and PLA in relation to CTRL indicate a possible role of sodium.

## INTRODUCTION

The high energy request of the organism during intensive efforts and the need for rapid anaerobic ATP resynthesis lead to the release of protons that decrease blood pH, and cause muscle acidification [1]. This occurs predominantly during high-intensity exercises and training programs, such as high-intensity interval training (HIIT) and/or high-intensity functional training (HIFT). It is well known that increased muscle acidification is one of the main suppressors of the ability to perform high-intensity exercise [2]. Moreover, HIIT/HIFT may influence homeostatic disturbances not only directly through the acid-base balance, but also indirectly affecting the various haematological and biochemical markers. However, post-exercise changes in these indices, especially haematological and muscle damage-specific markers, are equivocal.

White blood cell (WBC) count increases immediately after intense exercise [3, 4, 5]. Furthermore, despite an increase in WBC after HIIT and continuous aerobic exercise, WBC elevation is remarkably higher for HIIT than continuous aerobic exercise [6]. In addition, shifts in post-exercise haematological markers are related to an increase in the percentage of lymphocytes (LYM%) [4, 5, 6], monocytes (MON%) [4], and a decrease of granulocytes (GRA%) [6]. However, changes of the aforementioned markers are not always repeatedly recorded [6]. Similarly, ambiguous positions relate to exercise-induced changes of platelets (PLT), red blood cells (RBC) count and heamoglobin (HGB) concentration [3, 5, 7, 8].

Moreover, in most studies, relating to changes in blood biochemical markers, post-exercise increases in lactate dehydrogenase (LDH) [9], creatine kinase (CK) [3, 8, 9], alanine aminotransferase (ALT) and aspartate aminotransferase (AST) [10] activities or urea [11] and magnesium [7] concentrations have been shown, although changes in these indicators after intense efforts have not always been shown [10].

It is interesting to note that the aforementioned biochemical changes are sensitive to pH reductions induced by high-intensity exercise, which may affect the haematological profile in blood due to the role of haemoglobin as a buffer in the body [12] and increasing the risk of osmotic fragility of erythrocytes [13]. Furthermore, acidosis resulting from chronic kidney disease stimulates the activity of the ubiquitin-proteasome pathway and the formation of glucocorticoids, which contribute to the degradation of muscle proteins, and thus to an increase in blood urea concentration [14]. It would be reasonable to assume that this mechanism may also be reflected in acidosis resulting from highly intensive exercise.

While it is unwise to interfere with training and reduce the intensity of exercise (which may negatively affect adaptation and sports performance), an alternative to support the body’s buffering needs is nutritional and/or supplementation support. Alkalizing agents, such as sodium bicarbonate (NaHCO_3_), improve CrossFit-like performance [15] and have significant ergogenic effects [16] through increasing blood buffering capacity [17], which allows greater hydrogen ion (H^+^) binding, H^+^/lactate shuttle from working myocytes, and increases extra-cellular pH [16, 18].

NaHCO_3_ intake increases pH, through an elevation of bicarbonate ion (HCO_3_^−^) concentration in the blood, which may be beneficial in exercises heavily reliant on anaerobic metabolism [16]. Excessive sodium bicarbonate administration may induce severe alkalaemia, hypernatraemia and hypokalaemia [19]. However, in sport practice commonly supplemented doses ranging from 0.1 to 0.5 g · kg^−1^_BM_ NaHCO_3_ [16, 20] do not appear to be a cause for concern. The ideal supplementation method appears to be ingestion in a solution or gelatine capsules at a dose of ≥ 0.3 g · kg^−1^_BM_ NaHCO_3_ 60–180 min before exercise [16, 20, 21]. However, there are also reports that do not confirm this positive influence of NaH-CO_3_ [22, 23]. This may be due to the large diversity of the organism’s individual response to this agent and supplementation protocols [20, 24], or related to digestive system problems that can appear in some people [18, 25]. Due to lower tolerance, some athletes choose lower doses (~0.2 g · kg^−1^_BM_) and lengthening the time between NaHCO_3_ intake and exercise starting (~180 min), which may also have an ergogenic benefit [16, 20, 26]. On the other hand, Grgic et al. [20] reported the use of higher doses (0.4–0.5 g · kg^−1^_BM_) BM did not provide any additional benefits compared to the dose of 0.3 g · kg^−1^_BM_ NaHCO_3_. Therefore, despite performance effects, it is crucial to investigate the multidirectional impact of NaHCO_3_ on whole-body homeostasis during intense exercise.

It is not known to what extent acute HIIT/HIFT-induced changes in haematological and biochemical indicators are modulated by NaHCO_3_ supplementation. Extracellular buffering capacity elevation through NaHCO_3_ intake, proportionally to the administered dose, may enhance acid-base balance during efforts and could translate into changes in haematological and blood biochemical markers. The aim of this study was to verify the extent to which NaHCO_3_ would affect the acute changes of haematological and blood biochemical markers following intense exercise. We hypothesized that NaHCO_3_ would minimize exercise-induced changes in these markers in a dose-dependent manner.

## MATERIALS AND METHODS

### Participants

Thirty-four Cross-Fit-trained participants were initially enrolled in the study. Due to personal and professional reasons (business trips) 4 subjects (3 males (M)/1 female (F)) dropped-out of the study. Thirty athletes (16M/14F) completed the whole study protocol and were included in the analyses. The participants were regularly training HIFT in CrossFit-affiliated clubs in Poznań (“*Hangar*” and “*Stajnia CrossFit*”) and Szczecin (“*Papaj CrossFit*”) in Poland (Table 1).

**TABLE 1:** Somatic and physiological characteristics of participants training Cross-Fit^®^ (n = 30).

Characteristics	Mean ± SD	95% CI
Age [years]	30.6 ± 4.3	(29.0–32.2)
Body mass [kg]	73.8 ± 12.1	(69.3–78.3)
Fat-free mass [kg]	59.8 ± 11.5	(55.4–64.1)
Fat mass [%]	19.1 ± 5.5	(17.1–21.2)
Body height [cm]	175 ± 9	(172–178)

SD – standard deviation; CI – confidence interval

The inclusion criteria were: a valid and up-to-date medical certificate that confirmed the athlete’s ability to practice sports, good general health, at least 4 years of training experience and participation in a minimum of four CrossFit workout sessions a week. The exclusion criteria were: current injury, health-related contraindication, declared general feeling of being unwell and unwillingness to follow the study protocol.

The local institutional review board reviewed and approved the study protocol (Bioethics Committee at Poznan University of Medical Sciences, reference number: 1000/18 of 11 October 2018). The study protocol was also registered at *ClinicalTrials.Gov* (NCT03810404). All procedures were conducted in accordance with the ethical standards of the 1964 Declaration of Helsinki and written informed consent was obtained from all participants before their participation in the study began.

The required sample size was calculated a priori using a computer statistical package Statistica 13.3 (StatSoft, Inc., Tulsa, OK, USA) based on results of other studies [27, 28]. It was estimated that a sample size of 28 would be suitable for detecting a difference between blood pH measurements to obtain a power of approximately 90% (α = 0.05) in analysis of variance (ANOVA) with repeated measurements (RM) within factors.

### Study design

**FIG. 1:**
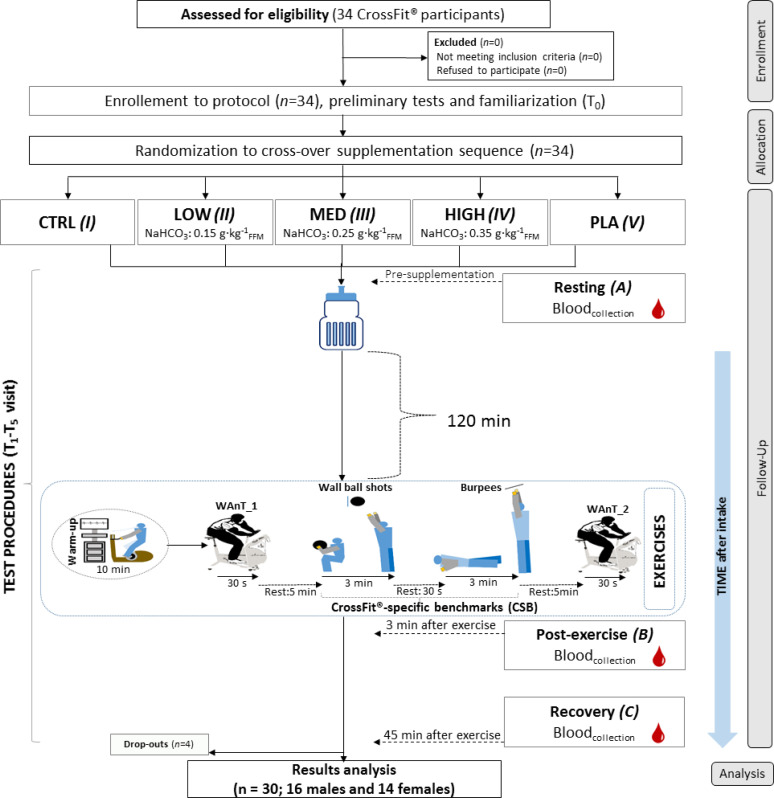
A flow chart of the study design. CTRL – control visit, without supplementation or placebo treatment; HIGH – visit with high NaHCO_3_ dose (0.35 g · kg^−1^_Fat-Free Mass_); LOW – visit with low NaHCO_3_ dose (0.15 g · kg^−1^_Fat-Free Mass);_ MED – visit with medium NaHCO_3_ dose (0.25 g · kg^−1^_Fat-Free Mass_); NaHCO_3_ – sodium bicarbonate; PLA – placebo; T_0_–T_5_ – numbers of study visits; WAnT – 30-s Wingate Anaerobic Test.

Supplementation of various NaHCO_3_ doses was evaluated in a randomized, multi cross-over, placebo (*PLA*)-controlled double-blind trial. The protocol of the study consisted of five visits (T1–T5) for each participant (Figure 1). In T1, a control (*CTRL*) phase was carried out where all tests without supplementation or *PLA* administration were performed. During subsequent visits (T2–T5), studies were conducted after three different doses of NaHCO_3_ and *PLA* intake depending on a randomization-based supplementation order.

All participants had substantial experience in performing the exercise procedures. They were also obligated to eat a standardized meal (containing 2 g of carbohydrates per kilogram of body mass (BM), 30 g of protein and at least 7 ml of water per kilogram of BM) two hours before each visit. Diet of the participants was monitored by an experienced dietician who ensured that athletes met all recommended criteria in terms of nutritional standards [29]. Further-more, participants did not follow any specific nutritional strategy and did not change any nutritional aspects during the study protocol.

The study visits (T1–T5) were separated by at least 7-days of washout.

### Supplementation

The NaHCO_3_ doses were provided relative to fat-free mass (FFM). The dose of supplemented NaHCO_3_ was considered as *LOW* (0.15 g·kg^−1^_FFM_ of NaHCO_3_), *MEDIUM* (0.25 g · kg^−1^_FFM_ of NaHCO_3_, *MED*) and *HIGH* (0.35 g · kg^−1^_FFM_ of NaHCO_3_). NaHCO_3_ in the form of powder was used (Alkala N, Sanum-Kehlbeck GmbH & Co. KG, Germany; containing 89.1% of NaHCO_3_, 9.9% of potassium bicarbonate, and 1.0% of sodium citrate). *PLA* preparation was provided in a similar powder form and contained sodium chloride in an equimolar amount of sodium as *MED* NaHCO_3_. All preparations were dissolved in approximately 750 mL of fluid (600 mL of water and 150 mL of orange juice). Juice was provided as a source of CHO to alleviate GI symptoms [18, 25, 30] and improve the taste of the preparations. Athletes were obligated to drink the preparations 2 h before the exercise tests within 5 min and were supervised by a member of the research team.

### Anthropometric measurements

Anthropometric measurements were performed according to recommendations as described previously [31]. Body mass and height were measured using a professional medical scale (WPT 60/150 OW, RADWAG, Radom, Poland). Body composition and total body water were assessed via bioelectric impedance, using Bodystat 1500MDD (Bodystat Inc., Douglas, UK).

### Exercise tests

The exercise battery started 2 h after NaHCO_3_/*PLA* intake (or fluid intake at *CTRL* – T1 visit). Each visit consisted of two classic 30-s Wingate tests (WAnT_1 and WAnT_2) separated by CrossFit-specific benchmarks: a) Wall Balls (performed for 3 min) and (after a 30 s transition break) b) Burpees (performed for 3 min). The Wall Balls task started exactly 5 min after the completion of WAnT_1, and WAnT_2 started 5 min after the completion of the Burpees task. The actual high-intensity active effort lasted a total of 7 min.

The exercise tests were always preceded by a standardized 10 min warm-up as previously described [31]. WAnTs were performed on a Monark 894E cycloergometer (Varberg, Sweden) and external loading was adjusted individually at 7.5% body weight [31]. During the CrossFit-specific benchmarks, the participants were instructed to perform as many repetitions of each exercise as they could during 3 min. Repetitions were accounted for, only if the participant completed a full range of motion required for each exercise. The only difference in CrossFit-specific benchmarks between female and male participants was the weight of the medicine ball during wall balls (6 and 9 kg for females and males) [32]. Participants were verbally encouraged to exert maximum effort throughout the exercise tests.

### Biochemical analyses

Capillary blood was obtained from the fingertip, at rest before supplementation (2 h before exercise), 3 min after (post-exercise) and 45 min (recovery) after exercise. Blood was collected according to the applicable and standardized procedures, from the finger of the nondominant hand using Medlance Red lancet-spike (HTL-Zone, Berlin, Germany) with a 1.5 mm blade and 2.0 mm penetration depth. A heparinized capillary sample (65 μl; Radiometer, Copenhagen, FFM 33 Denmark) was taken to determine pH using a blood gas analyzer (ABL90 FLEX, Radiometer, Copenhagen, Denmark). Additionally, 300 μl of capillary blood was collected in a Microvette CB 300 tube (Sarstedt, Nümbrect, Germany) containing EDTA dipotassium salt as an anticoagulant for WBC and their individual fractions (LYM%, MON%, GRA%), RBC, HGB, haematocrit (HCT) and PLT determination on a haematology analyzer (Mythic18, Orphèe, Geneva, Switzerland); and after plasma separation concentration of urea and creatinine, and ALT, AST, CK, and LDH activities on the Accent 220S automatic biochemical analyser (Cormay, Łomianki, Poland). Another 300 μl of capillary blood was collected in a Microvette CB 300 Z tube (Sarstedt, Nümbrect, Germany) with a clotting activator, in which the serum concentration of magnesium was marked also on the Accent 220S automatic biochemical analyzer.

Additionally, to avoid misinterpretation of blood parameter results due to different hydration status of participants on different visits, haematology indicators were related to the number of cellular components (WBC, RBC, HGB, PLT) and all biochemical parameters were converted using the haematocrit correction formula [33, 34].

### Statistical Analysis

Data are presented as the mean and standard deviation (± SD) and the 95% confidence interval for the mean (95% CI). The studied variables were checked for normal distribution using the Shapiro-Wilk test. For the comparison of the results between the five visits (T1–T5) and collection moment (rest, post-exercise and recovery), repeated measures ANOVA were performed for normally distributed data. For data that was not normally distributed, the Friedman ANOVA test was selected. Post-hoc (Tukey HSD test for parametric statistics and post hoc for Friedman for nonparametric statistics) analyses were then conducted for statistically significant data. The level of significance was set at p < 0.05. Statistical analysis was performed using a computer statistical package STATISTICA 13.3 (StatSoft, Inc., Tulsa, OK, USA).

## RESULTS

The resting (rest) values of all haematological, biochemical and blood gas measures did not differ significantly from one another across T1–T5.

### Blood pH value

**FIG. 2:**
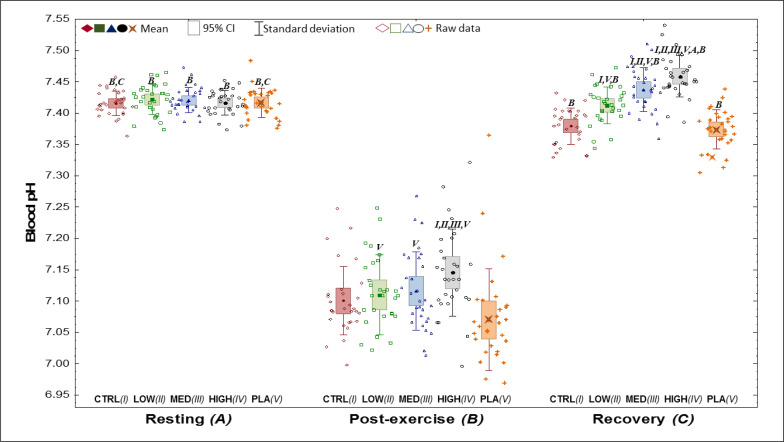
Resting (A), post-exercise (B) and recovery (C) pH values in the five phases (I-V). CI – confidence interval; CTRL – control visit, without supplementation or placebo treatment; HIGH – visit with high NaHCO_3_ dose ^−1^_Fat-Free Mass_); LOW – visit with low NaHCO_3_ dose (0.15 g · kg^−1^_Fat-Free Mass_); MED – visit with medium NaHCO_3_ dose (0.25 g · kg^−1^); NaHCO_3_– sodium bicarbonate; PLA – placebo. ^I,II,III,IV,V^The values of the phase, which number is in the superscript, are significantly lower than the values at which they are presented; ^A,B,C^The values of the moment, which letter is in the superscript, are significantly lower than the values at which they are presented.

All blood pH results are presented in Figure 2.

An exercise-induced decrease in pH (p < 0.001) was shown between post-exercise vs. resting values for each visit (*NaHCO*3*’s, CTRL, PLA*). However, in the recovery period, pH returned or even significantly exceeded (in *MED* and *HIGH*) the resting values after NaHCO_3_.

Differences were also visible after exercise (p < 0.001) between all NaHCO_3_ doses and *PLA*. Additionally, post-exercise and recovery blood pH was greater *HIGH* in comparison to the other visits. Furthermore, at recovery, pH was higher in *LOW* and *MED* than *CTRL* and *PLA* (p < 0.001).

### Blood Haematological Markers

All haematological results are presented in Table 2.

**TABLE 2:** Summary of the level of blood haematological parameters resting, post-exercise and recovery in the five visits of the study (n = 30).

Indicator	Measurement time	STUDY VISITS [Mean ± SD (95% CI)]					*p*-Value

		CTRL (I)	LOW (II)	MED (III)	HIGH (IV)	PLA (V)	
White blood cells [10^9^/L]	Resting (A)	7.7 ± 1.6	7.6 ± 1.9	7.6 ± 1.7	7.7 ± 1.7	7.4 ± 1.9	0.286
		(7.1–8.3)	(6.9–8.3)	(7.0–8.2)	(7.0–8.3)	(6.7–8.1)	
	
	Post-exercise (B)	13.2 ± 3.9^A,C^	13.1 ± 2.5^A,C^	13.1 ± 2.7^A,C^	13.3 ± 2.8^A,C^	13.2 ± 2.9^A,C^	0.746
		(11.8–14.6)	(12.2–14.1)	(12.1–14.1)	(12.2–14.3)	(12.1–14.3)	
	
	Recovery (C)	9.6 ± 2.8^II,III,IV,V,A^	8.1 ± 2.3	8.4 ± 2.6	8.0 ± 2.2	8.3 ± 2.6^A^	< **0.001**
		(8.6–10.7)	(7.2–9.0)	(7.4–9.4)	(7.1–8.8)	(7.4–9.3)	
	
	***p*-Value**	< **0.001**	< **0.001**	< **0.001**	< **0.001**	< **0.001**	

Lymphocytes [%]	Resting (A)	35.1 ± 7.0^C^	35.1 ± 6.7^C^	35.0 ± 7.4^C^	34.3 ± 6.6^C^	35.7 ± 7.1^C^	0.833
		(32.4–37.7)	(32.6–37.6)	(32.2–37.7)	(31.8–36.7)	(33.0–38.3)	
	
	Post-exercise (B)	44.1 ± 8.2^A,C^	46.4 ± 7.5^A,C^	47.1 ± 6.4^A,C^	44.9 ± 8.7^A,C^	47.0 ± 9.8^A,C^	**0.048** *
		(41.0–47.2)	(43.6–49.2)	(44.7–49.5)	(41.7–48.1)	(43.3–50.6)	
	
	Recovery (C)	23.0 ± 7.7	28.3 ± 8.2^I^	28.8 ± 9.2^I^	28.6 ± 9.4^I^	28.3 ± 10.2^I^	**0.004**
		(20.2–25.9)	(25.3–31.4)	(25.4–32.3)	(25.1–32.1)	(24.5–32.1)	
	
	***p*-Value**	< **0.001**	< **0.001**	< **0.001**	< **0.001**	< **0.001**	

Monocytes [%]	Resting (A)	6.7 ± 1.7	6.8 ± 1.9	7.2 ± 1.8	7.1 ± 2.3	6.8 ± 2.3	0.467
		(6.1–7.4)	(6.1–7.5)	(6.5–7.9)	(6.2–7.9)	(6.0–7.7)	
	
	Post-exercise (B)	7.2 ± 1.8	7.5 ± 1.6^A,C^	7.8 ± 1.7	7.5 ± 1.6	7.4 ± 1.3^A,C^	0.200
		(6.5–7.9)	(6.9–8.1)	(7.1–8.4)	(6.9–8.1)	(6.9–7.9)	
	
	Recovery (C)	6.1 ± 2.2	6.7 ± 1.7	7.5 ± 2.6^I^	7.4 ± 2.9	6.7 ± 1.9	**0.012**
		(5.3–6.9)	(6.1–7.3)	(6.5–8.4)	(6.3–8.5)	(6.0–7.4)	
	
	***p*-Value**	< **0.001**	< **0.001**	0.209	0.121	**0.001**	

Granulocytes [%]	Resting (A)	58.2 ± 7.7^B^	58.1 ± 7.1^B^	57.8 ± 7.6^B^	57.5 ± 7.9^B^	58.7 ± 6.9^B^	0.714
		(55.4–61.1)	(55.4–60.8)	(55.0–60.7)	(56.1–61.2)	(54.6–60.5)	
	
	Post-exercise (B)	48.7 ± 9.0	46.1 ± 7.8	45.2 ± 6.8	47.6 ± 9.2	45.6 ± 10.4	**0.037** *
		(45.3–52.0)	(43.2–49.0)	(42.6–47.7)	(44.1–51.0)	(41.7–49.5)	
	
	Recovery (C)	70.9 ± 8.9^II,III,IV,V,A,B^	65.0 ± 8.4^A,B^	63.7 ± 9.9^A,B^	64.0 ± 10.1^A,B^	65.0 ± 10.5^A,B^	< **0.001**
		(67.6–74.2)	(61.8–68.1)	(60.0–67.4)	(60.2–67.7)	(61.1–68.9)	
	
	***p*-Value**	< **0.001**	< **0.001**	< **0.001**	< **0.001**	< **0.001**	

Red blood cells [10^12^/L]	Resting (A)	5.96 ± 0.59^B^	5.94 ± 0.59^B^	5.92 ± 0.55^B,C^	5.91 ± 0.53^B,C^	5.90 ± 0.55^B^	0.119
		(5.74–6.19)	(5.72–6.16)	(5.71–6.12)	(5.71–6.10)	(5.70–6.11)	
	
	Post-exercise (B)	5.88 ± 0.59^III^	5.86 ± 0.60	5.81 ± 0.54	5.82 ± 0.53	5.83 ± 0.55	**0.033**
		(5.66–6.10)	(5.63–6.08)	(5.61–6.01)	(5.62–6.02)	(5.62–6.03)	
	
	Recovery (C)	5.96 ± 0.60^III,IV,V,B^	5.94 ± 0.59^B^	5.88 ± 0.53^B^	5.88 ± 0.52^B^	5.89 ± 0.56^B^	**0.007**
		(5.74–6.18)	(5.72–6.16)	(5.68–6.08)	(5.69–6.08)	(5.68 ± 6.10)	
	
	***p*-Value**	< **0.001**	< **0.001**	< **0.001**	< **0.001**	< **0.001**	

Haemoglobin [mmol/L]	Resting (A)	10.84 ± 0.40	10.63 ± 0.48^B,C^	10.75 ± 0.45^B,C^	10.75 ± 0.48^B,C^	10.63 ± 0.46^B,C^	0.076
		(10.69–10.98)	(10.45–10.81)	(10.58–10.91)	(10.57–10.94)	(10.45–10.80)	
	
	Post-exercise (B)	10.56 ± 0.47^II,V^	10.29 ± 0.54	10.35 ± 0.51	10.43 ± 0.62	10.35 ± 0.52	**0.004**
		(10.38–10.74)	(10.09–10.49)	(10.16–10.54)	(10.20–10.66)	(10.16–10.54)	
	
	Recovery (C)	10.71 ± 0.37^II,B^	10.46 ± 0.55^B^	10.51 ± 0.50^B^	10.56 ± 0.60^B^	10.47 ± 0.53^B^	**0.015**
		(10.57–10.84)	(10.26–10.67)	(10.32–10.69)	(10.33–10.78)	(10.27–10.66)	
	
	***p*-Value**	< **0.001**	< **0.001**	< **0.001**	< **0.001**	< **0.001**	

Platelets [10^9^/L]	Resting (A)	269 ± 95	280 ± 88	273 ± 89	272 ± 78	279 ± 97	0.081
		(234–305)	(247–313)	(240–306)	(243–301)	(242–315)	
	
	Post-exercise (B)	302 ± 114	319 ± 113^A,C^	308 ± 90^A,C^	302 ± 93^A,C^	307 ± 107^A^	0.368
		(259–344)	(277–361)	(275–342)	(268 ± 337)	(267–347)	
	
	Recovery (C)	264 ± 97	282 ± 94	288 ± 86	267 ± 82	288 ± 92	0.061
		(228–300)	(247–317)	(256–320)	(237–298)	(254–322)	
	
	***p*-Value**	0.131	**0.001**	< **0.001**	**0.002**	**0.001**	

CI – confidence interval; CTRL – control visit, without supplementation or placebo treatment; HIGH – visit with high NaHCO_3_ dose (0.35 g · kg^−1^_Fat-Free Mass_); LOW – visit with low NaHCO_3_ dose (0.15 g · kg^−1^_Fat-Free Mass_); MED – visit with medium NaHCO_3_ dose (0.25 g · kg^−1^_Fat-Free Mass_); NaHCO_3_ – sodium bicarbonate; PLA – placebo; SD – standard deviation

I,II,III,IV,V The values of the phase, which number is in the superscript, are significantly lower than the values at which they are presented

A,B,C The values of the moment, which letter is in the superscript, are significantly lower than the values at which they are presented

* The post-hoc test did not show any significant difference between the terms

**TABLE 3:** Summary of the level of blood biochemical parameters resting, post-exercise and recovery in the five visits of the study (n = 30).

Indicator	Measurement time	STUDY VISITS [Mean ± SD (95% CI)]					*p*-Value

		CTRL (I)	LOW (II)	MED (III)	HIGH (IV)	PLA (V)	
Urea [mmol/L]	Resting (A)	7.2 ± 2.0^B^	7.3 ± 2.1^B,C^	7.2 ± 2.0^B^	7.0 ± 1.8^B^	7.2 ± 1.9^B^	0.955
		(6.5–8.0)	(6.5–8.0)	(6.4–7.9)	(6.4–7.7)	(6.5–7.9)	
	
	Post-exercise (B)	7.0 ± 1.9	6.6 ± 1.7	6.4 ± 1.7	6.6 ± 1.8	6.6 ± 1.6	0.455
		(6.3–7.7)	(6.0–7.3)	(5.8–7.0)	(5.9–7.2)	(6.0–7.2)	
	
	Recovery (C)	7.3 ± 1.9^B^	7.0 ± 1.9^B^	6.8 ± 1.8^B^	7.0 ± 1.9^B^	7.1 ± 1.8^B^	0.621
		(6.6–8.0)	(6.3–7.7)	(6.2–7.5)	(6.3–7.7)	(6.4–7.7)	
	
	***p*-Value**	< **0.001**	< **0.001**	< **0.001**	< **0.001**	< **0.001**	

Magnesium [mmol/L]	Resting (A)	1.01 ± 0.14	0.97 ± 0.13	1.01 ± 0.16	1.01 ± 0.15	0.99 ± 0.11	0.455
		(0.96–1.07)	(0.92–1.02)	(0.95–1.07)	(0.96–1.07)	(0.95–1.03)	
	
	Post-exercise (B)	1.04 ± 0.14	1.00 ± 0.12	1.01 ± 0.13	0.99 ± 0.14	1.00 ± 0.12	0.178
		(0.98–1.09)	(0.95–1.05)	(0.96–1.06)	(0.94–1.04)	(0.95–1.04)	
	
	Recovery (C)	1.15 ± 0.19^IV,A,B^	1.09 ± 0.14^A,B^	1.11 ± 0.16^A,B^	1.08 ± 0.16^A,B^	1.09 ± 0.14^A,B^	**0.034**
		(1.08–1.22)	(1.03–1.14)	(1.06–1.17)	(1.02–1.14)	(1.04–1.15)	
	
	***p*-Value**	< **0.001**	< **0.001**	< **0.001**	**0.005**	< **0.001**	

Alanine aminotransferase [U/L]	Resting (A)	32.4 ± 10.4	32.8 ± 13.6	31.4 ± 9.5	33.2 ± 13.8	31.8 ± 10.4	0.865
		(28.5–36.3)	(27.7–37.9)	(27.8–34.9)	(28.1–38.4)	(27.9–35.7)	
	
	Post-exercise (B)	34.6 ± 10.6^A^	35.2 ± 14.7^A,C^	32.5 ± 9.6^A^	35.9 ± 14.3^A^	34.5 ± 11.3^A^	0.610
		(30.7–38.6)	(29.7–40.7)	(29.0–36.1)	(30.6–41.3)	(30.3–38.7)	
	
	Recovery (C)	34.1 ± 10.2^A^	34.2 ± 13.5^A^	32.7 ± 9.7^A^	35.3 ± 14.3^A^	33.2 ± 10.6^A^	0.272
		(30.3–37.9)	(29.1–39.2)	(29.0–36.3)	(30.0–40.7)	(29.2–37.1)	
	
	***p*-Value**	< **0.001**	< **0.001**	< **0.001**	< **0.001**	< **0.001**	

Aspartate aminotransferase [U/L]	Resting (A)	37.1 ± 11.2	34.9 ± 9.8	33.9 ± 7.1	34.3 ± 10.5	33.6 ± 7.4	0.378
		(32.9–41.3)	(31.2–38.5)	(31.3–36.6)	(30.4–38.2)	(30.8–36.3)	
	
	Post-exercise (B)	39.6 ± 12.5^A^	36.6 ± 10.9^A^	35.2 ± 7.3^A^	36.0 ± 11.1^A^	35.7 ± 9.2^A^	0.348
		(34.9–44.3)	(32.6–40.7)	(32.5–38.0)	(31.9–40.1)	(32.3 ± 39.1)	
	
	Recovery (C)	38.9 ± 12.7^A^	36.1 ± 10.8	36.0 ± 10.3	36.4 ± 11.7^A^	35.3 ± 7.8	0.218
		(34.1–43.6)	(32.1–40.1)	(32.2–39.8)	(32.0–40.8)	(32.4–38.2)	
	
	***p*-Value**	< **0.001**	**0.006**	**0.014**	< **0.001**	**0.026**	

Creatine kinase [U/L]	Resting (A)	409.3 ± 360.6	346.3 ± 259.4	388.0 ± 339.2	361.7 ± 255.4	324.2 ± 238.7	0.615
		(274.7–543.9)	(249.5–443.2)	(261.3–514.6)	(266.4–457.1)	(235.0–413.3)	
	
	Post-exercise (B)	462.9 ± 414.6^A^	389.5 ± 281.7^A^	427.6 ± 369.5^A^	404.1 ± 282.2^A^	372.3 ± 258.9^A^	0.371
		(308.1–617.8)	(284.3–494.7)	(289.6–565.6)	(298.7–509.5)	(275.6–469.0)	
	
	Recovery (C)	472.3 ± 441.2^A^	392.3 ± 282.0^A^	441.9 ± 379.1^A^	405.8 ± 278.3^A^	374.8 ± 257.3^A^	0.471
		(307.6–637.1)	(287.0–497.6)	(300.4–583.5)	(301.8–509.7)	(278.7–470.9)	
	
	***p*-Value**	< **0.001**	< **0.001**	< **0.001**	< **0.001**	< **0.001**	

Lactate dehydrogenase [U/L]	Resting (A)	417 ± 75	407 ± 80	412 ± 50	400 ± 57	420 ± 79	0.731
		(389–445)	(377–436)	(394–431)	(378–421)	(391–450)	
	
	Post-exercise (B)	456 ± 81^A^	450 ± 91^A^	452 ± 51^A,C^	429 ± 64^A^	463 ± 95^A^	0.195
		(426–486)	(417–484)	(433–471)	(405–453)	(427–498)	
	
	Recovery (C)	442 ± 69	425 ± 82	446–122	420 ± 70^A^	440 ± 80	0.306
		(416–468)	(395–456)	(401–492)	(394–446)	(410–470)	
	
	***p*-Value**	**0.002**	**0.001**	< **0.001**	**0.003**	< **0.001**	

Creatinine [μmol/L]	Resting (A)	98.1 ± 16.4	98.8 ± 13.5	100.0 ± 13.0	98.2 ± 15.2	99.9 ± 13.3	0.351
		(92.0–104.2)	(93.7–103.8)	(95.2–104.9)	(92.5–103.9)	(95.0–104.9)	
	
	Post-exercise (B)	112.1 ± 20.7^A,C^	111.2 ± 15.9^A^	109.5 ± 14.8^A^	109.0 ± 15.9^A^	110.5 ± 19.0^A^	0.538
		(104.3–119.8)	(105.3–117.2)	(104.0–115.0)	(103.1–115.0)	(103.4–117.6)	
	
	Recovery (C)	107.8 ± 16.7^A^	112.7 ± 14.5^I,A^	111.9 ± 15.5^A^	110.7 ± 17.6^A^	112.9 ± 18.9^I,A^	**0.003**
		(101.5–114.0)	(107.3–118.1)	(106.2–117.7)	(104.1–117.2)	(105.8–120.0)	
	
	***p*-Value**	< **0.001**	< **0.001**	< **0.001**	< **0.001**	< **0.001**	

CI – confidence interval; CTRL – control visit, without supplementation or placebo treatment; HIGH – visit with high NaHCO_3_ dose (0.35 g · kg^−1^_Fat-Free Mass_); *LOW* – visit with low NaHCO_3_ dose (0.15 g · kg^−1^_Fat-Free Mass_); MED – visit with medium NaHCO_3_ dose (0.25 g · kg^−1^_Fat-Free Mass_); NaHCO_3_ – sodium bicarbonate; PLA – placebo; SD – standard deviation

I,II,III,IV,V The values of the phase, which number is in the superscript, are significantly lower than the values at which they are presented

A,B,C The values of the moment, which letter is in the superscript, are significantly lower than the values at which they are presented

There were differences at recovery between visits for almost all haemotological measures (out of PLT count). For RBC count and HGB concentration, these differences were also apparent at post-exercise. WBC count (p < 0.001) and GRA% (p < 0.001) were higher in *CTRL* than all other study visits. Significant differences were shown for percentage of LYM (p = 0.004). MON% was higher only for *MED* vs. *CTRL* (p = 0.012). In the case of RBC count at post-exercise, *CTRL* was higher than *MED* (p = 0.033) and at recovery, *CTRL* was higher than *MED, HIGH* and *PLA* (p = 0.007). HGB concentration post-exercise was significantly higher in *CTRL* vs. *LOW* and *PLA* (p = 0.004), and at recovery *CTRL* was higher than *LOW* (p = 0.015). Further-more, an increase at post-exercise vs. resting values were shown in all study visits for: “WBC count and LYM%, while GRA%, RBC count and HGB concentration were decreased. Post-exercise MON% was greater vs. resting and recovery values in *LOW* (p < 0.001) and *PLA* (p = 0.001). PLT count was higher for all doses of NaHCO_3_ (*LOW*, p = 0.001; *MED*, p < 0.001; *HIGH*, p = 0.002) at post-exercise in comparison to resting and recovery values, and *PLA* post-exercise vs. rest (p = 0.001).

Comparison between resting and recovery values showed differences for all study visits only for LYM%, GRA% and HGB concentration. WBC count had higher recovery values in *CTRL* (p < 0.001) and *PLA* (p < 0.001), but not any NaHCO_3_ visit. For MON%, decreased values at recovery vs. rest were shown only in *CTRL* (p < 0.001), and for RBC count in *MED* (p < 0.001) and *HIGH* (p < 0.001).

WBC count and LYM% were decreased (p < 0.001) at recovery vs. post-exercise in all visits, while there was an increased %GRA, RBC count and haemoglobin concentration (p < 0.001). Additionally, MON% was lower at recovery vs. post-exercise in *CTRL, LOW* and *PLA*, as was PLT count in *LOW, MED* and *HIGH*.

### Blood Biochemical Markers

All biochemical results are presented in Table 3.

There were significant differences between visits only for magnesium and creatinine concentration at recovery. Blood magnesium concentration was higher in *CTRL* vs. *HIGH* (p = 0.034), while creatinine concentration was lower in *CTRL* vs. *LOW* and *PLA* (p = 0.003).

There was an increase at post-exercise vs. rest in all study visits for ALT, AST, CK, and LDH, and creatinine, while urea concentrations were decreased. There were no significant differences for magnesium concentration.

Some of the exercise-induced biochemical markers returned to their resting values after a short period of recovery (except magnesium and creatinine concentrations, and ALT and CK activities). More-over, in comparison to resting values, lower urea concentration (p < 0.001) was observed only in *LOW*, while higher AST occurred in *HIGH* (p < 0.001) and *CTRL* (p < 0.001). LDH was higher at recovery than at rest in *HIGH* (p = 0.003).

Urea and magnesium concentration were increased (all p < 0.001) at recovery vs. post-exercise in all visits. Additionally, lower ALT in *LOW* (p < 0.001), creatinine concentration in *CTRL* (p < 0.001) and LDH in *MED* (p < 0.001) were shown.

## DISCUSSION

To our knowledge, this study is the first to assess different NaH-CO3 dose supplementation-induced changes for haematological and biochemical indicators at rest, immediately after high-intensity exercises and after a 45-min recovery period. Undertaking such research could be extremely important to broaden the definition of the effect of this ergogenic agent to human (athlete) metabolism.

Resting values of pH and all haematological and biochemical blood indices did not differ across the five study visits (*CTRL, NaH-CO*3*’s, PLA*), which proves the homogeneity of the group for the studied indicators. This is an important aspect of research in order to properly compare the studied variables.

Post-exercise changes in blood pH values after taking different NaHCO_3_ doses compared to *CTRL* and *PLA*, were improved and consistent with those reported so far in the literature [27, 28, 30, 35]. The higher the NaHCO_3_ dose, the lower the post-workout acidification of the organism and more effective recovery of the index values back to resting values. Such an improvement in regeneration, acidbase balance indicators, when using NaHCO_3_ before exercise was also presented in the work by Siegler et al. [36] and Mündel [37]. On the other hand, taking NaHCO_3_ after exercise did not accelerate acid-base balance recovery, as indicated by Gurton et al. [38]. Thus, it seems clear that the ingestion of NaHCO_3_ has to be performed before efforts and preferably according to specific absorption time [16].

Interestingly, post-exercise WBC counts returned to baseline values faster with all NaHCO_3_ doses than with *CTRL* or *PLA*. However, these results did not have a direct impact on individual leukocyte subpopulations. The percentage values of LYM and GRA, despite differences between the *CTRL* phase and the remaining visits of study, do not indicate any effect of NaHCO_3_ intake. In contrast, MON% remained stable throughout the study period with *MED* and *HIGH* NaHCO_3_ supplementation doses. Such differences might be explained by the fact that the WBC count increases to counter exercise-induced inflammation [39]. Moreover, acidification of the body contributes to such an increase in inflammation [40]. Thus, if athletes supplement alkaline agents before exercise (e.g. with NaHCO_3_), inflammation may not be aggravated by acidosis and blood leukocytes count will be able to return to the resting state faster. Furthermore, extracellular buffering support may also hamper low blood pH-induced increase of the concentration of magnesium (Mg), due to the release of magnesium ions from complexes with proteins (mainly albumin), which, while buffering the blood, replace Mg with hydrogen (H^+^) ions [41].

No effect of NaHCO_3_ supplementation was noted for the HGB concentration and PLT count. Nevertheless, a relationship between NaHCO_3_ *MED* or *HIGH* dose supplementation and the lower RBC count between recovery *vs.* resting values was found. This is a surprising result as reducing the acidity of the organism should reduce the risk of osmotic fragility of erythrocytes [42] and thus more RBC with increased buffering capacity would be expected. However, the mechanism of this phenomenon (a decreased of RBC count after NaHCO_3_ supplementation or metabolic alkalosis) is unknown and according to our literature review no research has been carried out in this respect.

Physical effort, especially of a high intensity nature, leads to substantial changes in the activity of intramuscular enzymes, especially CK and LDH [43]. The elevated level of these indicators persists for a long time after finishing intensive exercise and their return to reference values may last even several days [44]. Alkalizing factors, i.e. NaHCO_3_, should hypothetically protect muscles against increased damage by hydrogen ions generated during intense exercise. It should be noted that physical efforts also lead to an increase in body temperature, which can additionally damage cells. Interestingly, studies on cell cultures indicate that the presence of NaHCO_3_ combining with vitamin C protects them from heat damage by increasing their antioxidant capacity [45]. In addition, studies in horses show that the administration of NaHCO_3_ reduces the symptoms of rhabdomyolysis [46]. After adding this supplement to the diet, the activity of muscle enzymes such as CK and AST were significantly reduced [46]. Similar results should be expected in athletes, although we did not show this in the current study as there was no relationship between the dose of NaHCO_3_ and their protective role against muscle fibers. However, it should be underlined that no studies have been conducted in this aspect. In addition, our investigation indicated that NaHCO3 does not affect the urea concentration in blood which confirm similar observations in animal (rats) *in vivo* studies [47].

It could also be assumed that highly-trained athletes may have a strengthened buffering capacity potential, while less trained ath-letes seems to be more dependent on additional NaHCO_3_ supple-mentation [48]. In our work, studied participants had substantial training experience (at least 4 years of training experience), so the lack of changes of muscle metabolites and enzymes activity may be the result of their adaptation to HIFT effort.

The strength of the described research is related primarily to the multi-crossover research design and novelty of supplementation of various FFM-adjusted doses in each participant. Much attention was also paid to compliance and control of the protocol, and use of rec-ommended research methods allowing for biochemical monitoring and induction of innovatory divided high-intensity exercises procedure. It is important that the haematological and biochemical indica-tors determined constitute a kind of novelty in this matter. In addition, they were attended by people who train CrossFit on a daily basis, so the efforts established in the test protocol were familiar to them. It is also important to note that the participants of the research were highly involved in it and received feedback at the end, which increased their compliance and awareness of supplementation with NaHCO_3_.

Interestingly, it could be suggested that the observed similarities in analyzed results could be linked to the equimolar amount of sodium in NaHCO_3_ and *PLA* which should be confirmed in further studies.

Finally, the presented study brings a lot of new and important scientific and practical approaches. It seems important to monitor the influence of bicarbonate not only on acid-base balance, but also on other blood measures (haematological and biochemical).

## CONCLUSIONS

The present study confirms the positive and dose-dependent alkalizing effect of NaHCO_3_ after exercise and during a short term recovery period which may be desirable in terms of effective sport practice. The use of NaHCO_3_ supplementation has a similar effect on haema-tological markers as a placebo apart from MON%, or WBC count, where a beneficial effect of supplementation on the faster return of the above-mentioned indicators to resting values was observed. Nevertheless, supplementation with NaHCO_3_ did not affect the post-exercise and recovery efficiency changes of the blood biochemical indicators in any way. The role of sodium intake in this process warrants further studies.

## Data Availability

The data that support the findings of this study are available from the corresponding author upon reasonable request.
